# Add-on treatment with Cerebrolysin improves clinical symptoms in patients with ALS: results from a prospective, single-center, placebo-controlled, randomized, double-blind, phase II study

**DOI:** 10.25122/jml-2023-0459

**Published:** 2023-12

**Authors:** Alfredo José Firstenfeld, Jorge Listorti, Nasser Jalaff, Claudia Patricia Loaiza Orozco, Francisco Navarrete Gosdenovich, Timo Schurr

**Affiliations:** 1 Servicio de Neurociencias, Universidad de Buenos Aires, Instituto Cardiológico Banfield, Buenos Aires, Argentina; 2 Department of Psychiatry, Psychotherapy, Psychosomatics and Medical Psychology, Division of Psychiatry I, Medical University Innsbruck, Innsbruck, Austria

**Keywords:** amyotrophic lateral sclerosis, clinical study, Cerebrolysin, riluzole, functional outcome

## Abstract

Amyotrophic lateral sclerosis (ALS) is a devastating and progressive neurodegenerative disease with limited treatment options available. Cerebrolysin is a drug candidate for the treatment of ALS because of its neuroprotective and neuroregenerative effects. We initiated a pilot clinical study of a combination of Cerebrolysin and riluzole to assess the therapeutic benefit of Cerebrolysin as an add-on treatment on clinical signs and symptoms in outpatients with ALS. Twenty patients with a clinically definitive diagnosis of ALS were enrolled and randomly assigned in a 1:1 ratio to receive Cerebrolysin or placebo. All patients received 50 mg of riluzole PO twice daily as a standard treatment. Patients in the Cerebrolysin group received intravenous injections of 10 mL of Cerebrolysin once daily, five days a week for the first month and three days a week for the next two months. Analysis of the ALS Functional Rating Scale – revised at Month 1 (primary outcome measure), showed a significant treatment effect in favor of Cerebrolysin with a 2.3-point improvement from baseline to Month 1 compared to a 0.9-point decrease in patients on placebo (P=0.005). The effect was maintained over the three-month study period, and the beneficial effect of Cerebrolysin over placebo was also evident in the secondary outcome measures. The safety analysis showed that the combination of riluzole and Cerebrolyisn was well tolerated. Our results demonstrate for the first time a significant clinical effect of Cerebrolysin in improving functional outcomes in patients with ALS and suggest that Cerebrolysin has potential as a novel therapeutic option for ALS.

## BACKGROUND

Amyotrophic lateral sclerosis (ALS), also known as Lou Gehrig's disease, is a devastating and progressive neurodegenerative disorder. It is characterized by the degeneration and death of motor neurons, leading to muscle weakness, atrophy, and eventually paralysis, which often causes death due to neuromuscular respiratory failure. It predominantly affects the upper motor neurons in the cortex as well as the lower motor neurons in the brain stem and spinal cord. The manifestation of the disease is widely variable in presentation, progression, and survival [1, 2]. Beyond motor neuron degeneration, ALS is also known to involve multiple other regions of the brain, including the frontal and temporal lobes, which contribute to cognitive and behavioral impairments in some patients [3]. ALS is a rare disease with an incidence of approximately 2 per 100,000 person-years and a prevalence between 6 and 9 per 100,000 people, with studies indicating a rising incidence [4-6].

Despite extensive research efforts, there is currently no cure for ALS. The available treatment options are limited, focusing mainly on symptom management and supportive care to enhance the quality of life for patients. Over 60 compounds with different modes of action have been evaluated in clinical trials [7], but only three have been approved for clinical use to date. The primary medication approved by the U.S. Food and Drug Administration (FDA) for ALS is riluzole [8, 9], which has shown modest benefits in terms of extending survival by reducing glutamate excitotoxicity. Edaravone, an antioxidant, has been approved for ALS treatment in some countries and has shown limited effects on disease progression [10]. Recently, the FDA approved AMX0035 [11], a fixed combination of sodium phenylbutyrate and taurursodiol, which is considered to mitigate mitochondrial dysfunction. These treatments only provide incremental benefits and do not address the underlying mechanisms driving the disease. The lack of more effective therapies for ALS underscores the urgent need for the development of novel treatment approaches. Various strategies are currently being developed using compounds that target different pathological processes involved in ALS, with the aim of slowing disease progression, preserving motor function, and improving overall quality of life [12].

Cerebrolysin is a peptide-based drug produced in a biotechnological process from purified porcine brain proteins. It consists of low-molecular-weight neuropeptides and amino acids and has been shown to exert neuroprotective, neuroregenerative, and neurotrophic properties. The pharmacological profile of Cerebrolysin in neurodegenerative diseases has been reviewed by Masliah and Diez-Tejedor [13]. In addition to its neuroregenerative effects, Cerebrolysin has been shown to effectively reduce glutamate-induced excitotoxicity [14-16], which is considered an important pathophysiological mechanism in ALS [17]. Cerebrolysin has also been shown to reduce neuronal damage induced by oxidative stress [15], another major contributor to ALS pathology [18]. In addition, *in vivo* studies have shown that Cerebrolysin prevents lesion-induced degeneration and death of motor neurons in rats [19, 20].

These properties make Cerebrolysin a candidate drug for the treatment of ALS. By promoting neuronal survival, protecting against neurodegeneration, and stimulating neuroregeneration, Cerebrolysin may have the potential as a novel therapeutic option to address the complex pathophysiological processes underlying ALS. To evaluate whether this promising pharmacological profile of Cerebrolysin translates into clinically relevant effects, we have initiated a pilot clinical study of a combination of Cerebrolysin and riluzole in patients with ALS.

## MATERIAL AND METHODS

### Study design

The objective of this investigator-initiated, prospective, single-center, placebo-controlled, randomized, double-blind pilot study was to evaluate the therapeutic benefit of Cerebrolysin on clinical signs and symptoms and its clinical safety in outpatients diagnosed with ALS. Patients who met all eligibility criteria were randomized 1:1 to the treatment groups according to an Excel-generated randomization list. Randomization was performed in blocks of ten patients. The investigator and all other study personnel, except for the study personnel who prepared the ready-to-use infusion solution of the study drug, were blinded until the database lock. The study was conducted at the Instituto Cardiológico Banfield, Buenos Aires, Argentina, between July 2021 and June 2022.

### Patient population

A total of 20 patients were enrolled and randomized to receive Cerebrolysin (verum group) or placebo (control group). Eligible patients were of either sex and at least 18 years of age, with a clinically definite diagnosis of ALS according to the El Escorial [21] and revised Airlie House [22] diagnostic criteria, limb onset and/or bulbar onset with pyramidal signs, and a Modified Ashworth Spasticity Scale [23] score of 3. Eligible patients were required to provide informed consent to participate in the study.

Patients with co-morbidities such as hepatic disease, renal failure or severe renal impairment, coronary disease, epilepsy, Parkinson’s disease, or dementia, and patients with any condition that might interfere with compliance with study procedures or influence outcome assessment were excluded from the study. Patients were also excluded if they were pregnant or breastfeeding, had participated in another interventional study within the previous two months, or had a contraindication to Cerebrolysin. Concomitant use of ginkgo biloba, erythropoietin, citicoline, and amantadine was also an exclusion criterion.

### Treatment

Patients were randomized in a 1:1 ratio to receive Cerebrolysin or placebo. Patients in the Cerebrolysin group received intravenous injections of 10mL Cerebrolysin once a day, five days a week for the first month, then three days a week for the next two months. Patients in the placebo group received the same treatment with 10 mL of normal saline. All patients received 50 mg of riluzole PO twice daily as a standard treatment for ALS. The patient was treated at home by a specialist nurse.

### Outcome measures

Clinical assessment of the patients was performed at baseline before initiation of treatment, and subsequently, safety and efficacy evaluations were performed at Month 1, Month 2, and the end of the treatment period at Month 3. The efficacy evaluation included the ALS Functional Rating Scale – revised (ALSFRS-R) to assess motor impairment and functional deterioration [24], the modified Ashworth Scale (MAS) to assess spasticity, and the Beck’s Depression Inventory-II (BDI-II) to assess depressive symptoms [25]. Gross motor skills were also assessed by measuring the time taken to walk four meters, the total distance walked within a set time frame, the count of knee bends reaching the opposite arm, and hand strength measures using a handheld dynamometer. Drug safety was assessed by documenting adverse events throughout the study.

### Primary and secondary endpoints

Functional impairment, measured by the change in ALSFRS-R score from baseline to Month 1, was the primary outcome measure for the study. Secondary endpoints were the ALSFRS-R change from baseline to Months 2 and 3, as well as the change from baseline in the BDI-II, the MAS, and the motor evaluations at Months 1, 2, and 3. To analyze safety, the number of adverse events and deaths in the two study groups was monitored throughout the study through visits and weekly telephone calls.

### Statistical analysis

All efficacy analyses were performed on the intention-to-treat (ITT) population, which included all randomized patients who received at least one dose of the study drug (Cerebrolysin/placebo) and completed at least one post-baseline assessment. Analysis was performed using SPSS version 27.0. Descriptive statistics were presented for the intention-to-treat population based on observed cases, as imputation methods for missing data were not applied due to the small sample size. The Mann-Whitney test was used to compare the mean differences between the verum and the placebo groups in the change from baseline to follow-up in each outcome parameter. To control for multiple testing, a fixed-sequence test (separately for each secondary outcome), as described by Bauer *et al*. [26], was used. An alpha level of 0.05 was used for each test, with an a priori test sequence stopping at the first non-significant result. For all endpoints, the sequence was as follows: change from baseline to one-month follow-up, change from baseline to two-month follow-up, and finally change from baseline to three-month follow-up. P values less than 0.05 were considered statistically significant.

## RESULTS

A total of 20 patients were enrolled and randomized. Two patients in the control group discontinued the study during or after the baseline visit and were removed from the ITT dataset due to missing post-baseline data. Subsequently, the ITT dataset consisted of eight patients in the placebo group and 10 patients in the verum group. Demographic and baseline characteristics were similar between the two groups (Table 1).

**Table 1:** Demographics and disease severity at baseline

Variable		Placebo (n=8)	Cerebrolysin (n=10)	Statistics	p-value
Age (mean, SD)		46.88±10.36	56.4±10.20	Z=-1.65, r=0.39	0.098
Gender (N, %)	Male female	7 (87.5%) 1 (12.5%)	8 (80%) 2 (20%)	χ^2^(1)=0.18, OR=1.75	0.671
Ethnicity (N, %)	Caucasian	8 (100%)	10 (100%)	-	-
Dominant hand (N, %)	right	8 (100%)	10 (100%)	-	-
ALSFRS-R (mean, SD)		29.75±9.29	30.60±9.51	Z=-0.490, r=0.12	0.624
BDI (mean, SD)		10.38±6.21	12.20±5.09	Z=-0.895, r=0.21	0.371
MAS (mean, SD)		4±0	4±0	-	-
Walking time – 4 m [sec]		10.15±6.51	8.76±5.94	Z=-0.471, r=0.11	0.637
Walking distance – 120 sec [m]		51.83±47.15	57.95±61.56	Z=-0.471, r=0.11	0.637

**Figure 1:**
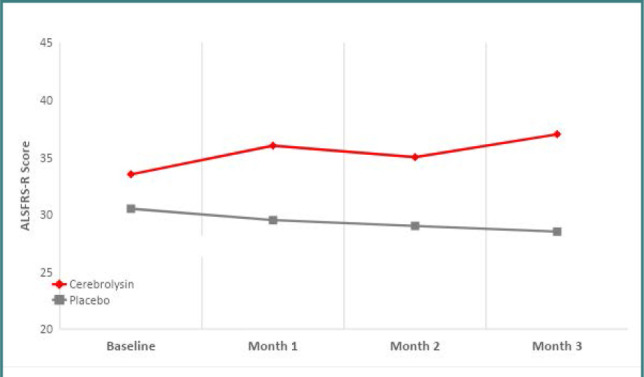
Development of the ALSFRS-R score over time

The primary efficacy analysis showed a significant treatment effect in favor of Cerebrolysin on the revised ALS Functional Rating Scale (ALSFRS-R). While Cerebrolysin patients improved by 2.3 points from baseline to Month 1, placebo patients deteriorated by 0.9 points. At least descriptively, patients on placebo continued to deteriorate over time due to the progressive nature of the disease, whereas patients on Cerebrolysin maintained and even slightly improved their functional level throughout the study period (Table 2, Figure 1).

**Table 2:** Primary efficacy analysis – functional outcome

ALSFRS-R Score	Placebo (n=8)		Cerebrolysin (n=10)		Statistics			
	Mean ± SD	Mean Rank	Mean ± SD	Mean Rank	U	Z	r	p-value^a^
Baseline – Month 1^b^	-0.88±1.64	5.56	2.3±2.1	12.65	8.500	-2.83	0.67	**0.005**
Baseline – Month 2	-1.63±1.41	6.75	-0.3±5.91	11.70	18.0	-1.97	0.46	**0.049**
Baseline – Month 3	-2.0±1.69	6.69	0.6±6.96	11.75	17.5	-2.01	0.47	**0.045**

a asymptotic significance (2-tailed); ^b^ pre-defined primary outcome parameter

The changes from baseline to Month 1 in the secondary efficacy parameters were consistent with the observed treatment benefit of Cerebrolysin (Table 3). Notably, the development of spasticity according to the MAS score over the study period was consistent with the results of the functional outcome (ALSFRS-R). Placebo patients showed no improvement in spasticity over three months (4±0 points at all study visits), with the affected limb being rigid in flexion or extension. In contrast, patients in the Cerebrolysin group improved continuously from 4±0 points at baseline to 2.90±0.32 at Month 3. Analyses of knee flexion were not performed as none of the patients were able to bend their knees.

**Table 3:** Secondary efficacy analysis

Variable	Placebo		Cerebrolysin		Statistics		
	Mean ± SD	Mean Rank	Mean ± SD	Mean Rank	U	Z	p-value^a^
**BDI^b^**							
Baseline – Month 1	1.75±2.55	13.19	-1.9±2.18	6.55	10.5	-2.66	**0.008**
Baseline – Month 2	3.88±4.49	11.88	0.5±5.54	7.60	21.0	-1.71	0.088
Baseline – Month 3	analysis stopped						
**MAS^b^**							
Baseline – Month 1	0±0	14.00	-0.9±0.32	5.90	4.0	-3.69	**<0.001**
Baseline – Month 2	0±0	14.50	-1.1±0.32	5.50	0.0	-4.0	**<0.001**
Baseline – Month 3	0±0	14.50	-1.1±0.32	5.50	0.0	-4.0	**<0.001**
**Walking time – 4 m [sec]**							
Baseline – Month 1^c^	0.32±0.48	11.58	-0.52±0.64	5.61	5.5	-2.54	**0.011**
Baseline – Month 2^d^	0.43±0.27	8.00	-0.10±1.08	6.14	15.0	-0.86	0.391
Baseline – Month 3	analysis stopped						
**Walking distance – 120 sec [m]^c^**							
Baseline – Month 1	-3.75±8.80	6.00	2.21±6.23	9.33	15.0	-1.42	0.157
Baseline – Month 2	analysis stopped						
Baseline – Month 3	analysis stopped						

a asymptotic significance (2-tailed); ^b^ n = 8 for placebo and n = 10 for Cerebrolysin; ^c^ n = 6 for placebo and n = 9 for Cerebrolysin; unable to walk n = 2 for placebo and n = 1 for Cerebrolysin; ^d^ n = 6 for placebo and n = 7 for Cerebrolysin; unable to walk n = 2 for placebo and n = 3 for Cerebrolysin

Similarly, descriptive analysis of right-hand grip strength showed that strength decreased in the placebo group from 46.00±24.46 kg at baseline to 42.43±20.49 kg, whereas it increased in the Cerebrolysin group from 50.00±18.37 kg to 53.44±23.93 kg. Essentially, similar results were obtained for left-hand grip strength. The safety analysis showed no significant or obvious differences in the number or nature of adverse events between the two study groups (Table 4).

**Table 4:** Adverse events

Treatment group	Adverse event term	SAE	Causality	Severity	Outcome
Cerebrolysin	Insomnia	no	unlikely	mild	recovered
Cerebrolysin	Covid-19	no	not related	mild	recovered
Cerebrolysin	Respiratory insufficiency	yes	not related	severe	recovered
Cerebrolysin	Respiratory insufficiency	yes	not related	severe	recovered
Placebo	Pneumonia	yes	Not related	severe	fatal

Three serious adverse events requiring hospitalization were reported: one case of fatal pneumonia in the placebo group and two cases of respiratory insufficiency in the Cerebrolysin group, both of which resolved and are considered well-known complications of the underlying disease. Overall, the addition of Cerebrolysin to riluzole was well tolerated and safe.

## DISCUSSION

Our results have demonstrated for the first time a significant clinical effect of Cerebrolysin in improving functional outcome, depressive symptoms, and spasticity in ALS patients for up to three months after the baseline assessment. In addition, we have shown for the first time that Cerebrolysin can be safely administered to ALS patients at a dose of 10 mL in combination with riluzole.

The primary outcome, functional impairment measured by the change from baseline to Month 1 in the ALSFRS-R score, as well as spasticity, showed a robust and significant improvement in the Cerebrolysin group compared to placebo, which was maintained throughout the three-month study period. There was an improvement in depressive symptoms and walking speed at one month, but the signal was lost after that. This may be because the total dose applied during Months 2 and 3 was lower than that applied during Month 1 in our study. This treatment regimen was chosen based on previous clinical trials with Cerebrolysin in other neurodegenerative and cerebrovascular diseases [27-30] and considering the manageability of home administration. In addition, a relatively low dose of 10mL per day was used, which may not represent the optimal treatment regimen for Cerebrolysin in ALS. Further optimization of the dose and treatment schedule will be required to achieve the best possible outcome.

Notably, patients in both groups were treated with riluzole, the gold standard treatment treatment for ALS. The additional clinical effect of Cerebrolysin was achieved in addition to the basic and well-documented effect of riluzole. At this stage, we are unable to determine whether Cerebrolysin had a treatment effect on ALS on its own or whether it acts synergistically to enhance the effect of riluzole. Studies of Cerebrolysin as a stand-alone treatment should be considered to answer this question.

It is important to note that, at least descriptively, patients on Cerebrolysin improved their functional score from baseline over the three-month study period, while patients on placebo worsened over the same period, in line with the progressive nature of the disease. Interestingly, the development of spasticity and grip strength in the dominant hand showed similar results, with improvement in the Cerebrolysin group over three months and deterioration in the placebo group, probably due to disease progression. This may indicate a stabilizing or even disease-modifying or neuro-regenerative effect of Cerebrolysin in ALS. Of course, a longer follow-up of one year or more would be needed to confirm this observation.

This study showed a significant improvement in spasticity in our patient population after treatment with Cerebrolysin. This is consistent with previous studies in patients with ischemic stroke, where a significant reduction in spasticity has been reported [31, 32]. Therefore, this may be a more general effect of Cerebrolysin on spastic symptoms not limited to ALS patients due to its neuroregenerative potential.

The small sample size, a major limitation of this study, did not allow analysis of certain subgroups, such as rapidly progressing patients. However, this study was designed as a pilot study to assess the feasibility and safety of a combination treatment with Cerebrolysin in a new indication. In any case, the clinical effect achieved despite the small sample size is remarkable.

## CONCLUSION

Our findings suggest that Cerebrolysin may have disease-modifying effects in ALS and has potential as a novel therapeutic option. However, the exact mechanisms by which Cerebrolysin exerts its effects in ALS are not fully understood. The combination of neurotrophic peptides and amino acids in Cerebrolysin may play a critical role in promoting motor neuron survival, ameliorating glutamate-induced excitotoxicity, modulating inflammatory processes, and promoting neuronal regeneration, all mechanisms closely associated with ALS pathogenesis. In addition, the potential effect of Cerebrolysin on the cognitive and behavioral symptoms associated with ALS highlights its potential as a promising treatment option for this complex disease. While our preliminary results for Cerebrolysin in ALS are encouraging, it is important to emphasize that further research is needed to confirm its clinical efficacy, optimize the therapeutic regimen, and shed more light on the exact mechanism of action in ALS. Overall, ALS remains a challenging disease with limited treatment options. However, recent advances in the understanding of the disease and the development of new therapeutic paradigms offer hope for the future. Further research into Cerebrolysin and other potential treatments has the potential to improve outcomes and quality of life for people with ALS.

## Data Availability

Further data is available from the corresponding author upon reasonable request.
